# P107 Recurrent renal stones may signal systemic disease: Sjögren’s syndrome-associated distal renal tubular acidosis

**DOI:** 10.1093/rap/rkag113.137

**Published:** 2026-11-03

**Authors:** Nishita Padmanabhan, Kottarathil Abraham, Rosalind Benson

**Affiliations:** NHS University Hospitals of Liverpool Group, Liverpool, United Kingdom; NHS University Hospitals of Liverpool Group, Liverpool, United Kingdom; NHS University Hospitals of Liverpool Group, Liverpool, United Kingdom

## Abstract

**Introduction:**

Recurrent nephrolithiasis may represent an under-recognised manifestation of systemic autoimmune disease. In Sjögren’s syndrome, distal renal tubular acidosis (dRTA) can lead to metabolic abnormalities and recurrent kidney stone formation, indicating underlying tubular dysfunction. We present a case of Sjögren’s syndrome with nephrolithiasis due to dRTA, further complicated by chronic pain and limited English proficiency, highlighting challenges in symptom control.

**Case description:**

A 35-year-old woman presented with recurrent episodes of severe right-sided flank pain that radiated to the groin, lasting up to 48 hours and recurring every few days. These episodes were accompanied by dysuria and the passage of “grit-like” material. The symptoms significantly impaired her functioning and necessitated multiple urological interventions, including ureteroscopy, laser lithotripsy, and ureteric stenting. The patient had very limited English proficiency, requiring interpreter support. She also reported a two-year history of inflammatory joint pain characterised by prolonged morning stiffness, intermittent swelling which was poorly responsive to opioid-based analgesia. She had Raynaud’s phenomenon, sicca symptoms and marked fatigue.

Rheumatological investigations revealed a positive ANA, rheumatoid factor, anti-Ro52 and weak-positive anti-Ro-60/SS-A antibodies. Immunoglobulins and CRP were within normal limits. ESR was elevated. Biochemical analysis showed persistent metabolic acidosis with preserved eGFR. A salivary gland ultrasound indicated sialadenitis-type changes. She was diagnosed with Sjögren’s disease and was started on hydroxychloroquine.

The combination of metabolic acidosis and recurrent nephrolithiasis raised suspicion for dRTA, which was subsequently confirmed by synchronous urine pH and venous bicarbonate level testing and stone analysis that demonstrated calcium apatite calculi. Sodium bicarbonate was prescribed to correct the acidosis.

The patient also experienced chronic pain, complicating the assessment of her response to hydroxychloroquine. Adherence to therapy was inconsistent, partly due to limited perceived symptomatic benefit and difficulty understanding the treatment’s preventive role. Ongoing care required coordinated input from rheumatology, nephrology, urology, and interpreter services.

**Discussion:**

This case underscores the complexity of Sjögren’s syndrome, particularly when organ-specific pathology coexists with significant symptom burden, chronic pain, and patient-related factors. Distal renal tubular acidosis is a recognised manifestation of this condition, resulting from impaired distal acid secretion. This leads to metabolic acidosis and an alkaline urinary environment that promotes stone formation. Acknowledging this biochemical pattern is crucial, as alkali therapy can potentially reduce the risk of recurrence.

Despite targeted treatment, this patient continued to experience persistent pain that impacted daily functioning. The symptoms were multifactorial, including inflammatory arthralgia and chronic pain, reflecting the well-documented discordance between objective findings and patient-reported outcomes in Sjögren’s syndrome.

Communication barriers further complicated the situation and may have affected the patient’s understanding of the diagnosis and treatment rationale, leading to challenges with adherence, particularly for sodium bicarbonate, whose benefits are preventive rather than immediate.

Effective management included reinforcing education about disease mechanisms, optimising analgesia while considering non-inflammatory factors, and adapting communication strategies to enhance understanding. Multidisciplinary collaboration was essential to address the renal, rheumatological, and urological aspects of care concurrently. This case highlights that achieving optimal care in Sjögren’s syndrome requires integrating pathophysiology, symptom management, and patient-centred communication to realise meaningful clinical outcomes.

**Key learning points:**

• Sjögren’s syndrome often manifests with extraglandular features that can significantly influence the overall clinical picture. Distal renal tubular acidosis should be considered in patients presenting with metabolic acidosis, stable renal function, and recurrent nephrolithiasis. Identifying this association facilitates the prompt initiation of alkali therapy, which can help decrease recurrence rates and prevent long-term renal complications.

• The symptom burden in Sjögren’s syndrome is frequently considerable and multifaceted. Patients may experience inflammatory, mechanical, and centrally mediated pain, as well as fatigue and diminished quality of life. These symptoms can persist even when laboratory parameters are stable, underscoring the limitations of reliance on objective indicators alone. Effective pain management necessitates a multimodal strategy.

• Limited proficiency in English may complicate shared decision-making and understanding of treatment options. Professional interpretation and repeated explanations are necessary to facilitate the patient’s engagement and compliance.

• Multidisciplinary collaboration remains important in managing complex cases of Sjögren’s syndrome. It ensures a comprehensive approach to organ involvement while treating symptom burden.

